# A Multifaceted Intervention to Improve the Quality of Care of Children in District Hospitals in Kenya: A Cost-Effectiveness Analysis

**DOI:** 10.1371/journal.pmed.1001238

**Published:** 2012-06-12

**Authors:** Edwine W. Barasa, Philip Ayieko, Susan Cleary, Mike English

**Affiliations:** 1 Kenya Medical Research Institute (KEMRI) Centre for Geographic Medicine Research – Coast, and Wellcome Trust Research Programme, Nairobi, Kenya; 2 Health Economics Unit, University of Cape Town, Cape Town, South Africa; 3 Department of Pediatrics, University of Oxford, Oxford, United Kingdom; Baylor College of Medicine, United States of America

## Abstract

**Background:**

To improve care for children in district hospitals in Kenya, a multifaceted approach employing guidelines, training, supervision, feedback, and facilitation was developed, for brevity called the Emergency Triage and Treatment Plus (ETAT+) strategy. We assessed the cost effectiveness of the ETAT+ strategy, in Kenyan hospitals. Further, we estimate the costs of scaling up the intervention to Kenya nationally and potential cost effectiveness at scale.

**Methods and Findings:**

Our cost-effectiveness analysis from the provider's perspective used data from a previously reported cluster randomized trial comparing the full ETAT+ strategy (*n* = 4 hospitals) with a partial intervention (*n* = 4 hospitals). Effectiveness was measured using 14 process measures that capture improvements in quality of care; their average was used as a summary measure of quality. Economic costs of the development and implementation of the intervention were determined (2009 US$). Incremental cost-effectiveness ratios were defined as the incremental cost per percentage improvement in (average) quality of care. Probabilistic sensitivity analysis was used to assess uncertainty. The cost per child admission was US$50.74 (95% CI 49.26–67.06) in intervention hospitals compared to US$31.1 (95% CI 30.67–47.18) in control hospitals. Each percentage improvement in average quality of care cost an additional US$0.79 (95% CI 0.19–2.31) per admitted child. The estimated annual cost of nationally scaling up the full intervention was US$3.6 million, approximately 0.6% of the annual child health budget in Kenya. A “what-if” analysis assuming conservative reductions in mortality suggests the incremental cost per disability adjusted life year (DALY) averted by scaling up would vary between US$39.8 and US$398.3.

**Conclusion:**

Improving quality of care at scale nationally with the full ETAT+ strategy may be affordable for low income countries such as Kenya. Resultant plausible reductions in hospital mortality suggest the intervention could be cost-effective when compared to incremental cost-effectiveness ratios of other priority child health interventions.

*Please see later in the article for the Editors' Summary*

## Abstract 2

A cost-effective analysis conducted by Edwine Barasa and colleagues estimates that a complex intervention aimed at improving quality of pediatric care would be affordable and cost-effective in Kenya.

## Editors' Summary

**Background:**

According to latest global estimates from UNICEF, 7.6 million children currently die every year before they reach five years of age. Half of these deaths occur in children in sub-Saharan Africa and tragically, most of these deaths are due to a few treatable and preventable diseases, such as pneumonia, malaria, and diarrhea, for which effective interventions are already available. In order to meet the target of the 4th Millennium Development Goal—which aims to reduce the under-five child mortality rate by two-thirds from 1990 levels by 2015—delivering these interventions is essential.

In Kenya, the under-five child mortality rate must be reduced by half from its 2008 level in order to meet the Millennium Development Goal (MDG) target and so improving the management of serious child illness might help achieve this goal. A study published last year in *PLoS Medicine* described such an approach and included the development and implementation of evidence-based clinical practice guidelines linked to health worker training, follow-up supervision, performance feedback, and facilitation in eight district hospitals in Kenya.

**Why Was This Study Done?:**

In the study mentioned above, the researchers compared the implementation of various processes of care in intervention and control hospitals at baseline and 18 months later and found that performance improved more in the intervention hospitals than in the control hospitals. However, while this strategy was effective at improving the quality of health care, it is unclear whether scaling up the approach would be a good use of limited resources. So in this study, the same researchers performed a cost-effectiveness analysis (which they conducted alongside the original trial) of their quality improvement intervention and estimated the costs and effects of scaling up this approach to cover the entire population of Kenya.

**What Did the Researchers Do and Find?:**

In order to perform the cost part of the analysis, the researchers collected the relevant information on costs by using clinical and accounting record reviews and interviews with those involved in developing and implementing the intervention. The researchers evaluated the effectiveness part of the analysis by comparing the implementation of their improved quality of care strategy as delivered in the intervention hospitals with the partial intervention as delivered in the control hospitals by calculating the mean percentage improvement in the 14 process of care indicators at 18 months. Finally, the researchers calculated the costs of scaling up the intervention by applying their results to the whole of Kenya—121 hospital facilities with an estimated annual child admission rate of 2,000 per facility.

The researchers found that the quality of care (as measured by the process of care indicators) was 25% higher in intervention hospitals than in control hospitals, while the cost per child admission was US$50.74 in intervention hospitals compared to US$31.1 in control hospitals. The researchers calculated that each percentage improvement in the average quality of care was achieved at an additional cost of US$0.79 per admitted child. Extrapolating these results to all of Kenya, the estimated annual cost of scaling up the intervention nationally was US$3.6 million, about 0.6% of the annual child health budget in Kenya.

**What Do These Findings Mean?:**

The findings of this cost-effectiveness analysis suggests that a comprehensive quality improvement intervention is effective at improving standards of care but at an additional cost, which may be relatively cost effective compared with basic care if the improvements observed are associated with decreases in child inpatient mortality. The absolute costs for scaling up are comparable to, or even lower than, costs of other, major child health interventions. As the international community is giving an increasing focus to strengthening health systems, these findings provide a strong case for scaling up this intervention, which improves quality of care and service provision for the major causes of child mortality, in rural hospitals throughout Kenya and other district hospitals in sub-Saharan Africa.

**Additional Information:**

Please access these Web sites via the online version of this summary at http://dx.doi.org/10.1371/journal.pmed.1001238.

- The researchers' original article appeared in *PLoS Medicine* in 2011: Ayieko P, Ntoburi S, Wagai J, Opondo C, Opiyo N, et al. (2011) A Multifaceted Intervention to Implement Guidelines and Improve Admission Paediatric Care in Kenyan District Hospitals: A Cluster Randomised Trial. PLoS Med 8(4): e1001018. doi:10.1371/journal.pmed.1001018
- The IDOC Africa provides further information on the ETAT+ strategy
- The World Health Organization (WHO) provides information on MDG 4, including strategies to reduce global child mortality) and the WHO pocket-book “Hospital care for children” includes guidelines for the management of common but serious childhood illnesses in resource-limited settings
- UNICEF www.unicef.org also publishes information on global child mortality rates and the Countdown to 2015 website tracks coverage levels for health interventions proven to reduce child mortality

## Introduction

An estimated 7.6 million children die globally every year before the age of five [1]. 99% of these deaths occur in developing countries; 50% in sub-Saharan Africa [2]. Most of these deaths are due to a few treatable and preventable diseases, for which effective interventions are already available [3],[4]. Delivering these interventions is essential to achieving the 4th Millennium Development Goal (MDG), which aims to reduce the under-five mortality rate by two-thirds by 2015. In Kenya, the under-five mortality rate has to be reduced by 50% from its 2008 level to meet the MDG target. Improving case management of serious illness might help achieve this goal [5],[6], and we have recently described one possible approach to this in Kenyan district hospitals [7]. That approach included the development and implementation of evidence-based clinical practice guidelines (CPGs) linked to health worker training, follow-up supervision, performance feedback, and facilitation (for brevity referred to as the ETAT+ strategy) [8],[9]. However, while the strategy was effective, would scaling up the approach be a good use of limited resources? Addressing this question demands a rigorous evaluation of costs and consequences with such data used to estimate the costs and effects of scaling up the intervention to reach the population in need. This paper presents a cost-effectiveness analysis performed alongside the previously reported cluster randomized trial of the effects of the ETAT+ strategy. We also present an assessment of the costs of scaling up the intervention to the national level and speculate on, using a simple model that assumes the strategy reduces inpatient mortality, the possible costs per disability adjusted life year (DALY) averted.

## Methods

### Study Design

This was a cost-effectiveness analysis alongside a cluster randomized controlled trial (cRCT). The time horizon selected was 18 mo (September 2006–April 2008), which was the period during which the intervention was implemented and evaluated. Costing took a provider's perspective. While this is often considered narrow [10],[11], for the purpose of this analysis, we considered it sufficient as it encompasses the relevant range of costs and effects of interest to policy makers responsible for budgeting and planning for scale-up in Kenya. To account for differential timing and time preference, we discounted costs and outcomes using a 3% annual discount rate [12]. Costs were adjusted for inflation using gross domestic product (GDP) deflators for Kenya [13] and are valued and presented in 2009 US$. Effects are measured in terms of process indicators of quality of care that include important measures of child assessment, diagnosis, classification, and treatment on admission. Probabilistic sensitivity analysis using Monte Carlo simulation was used to assess the impact of uncertainty around hotel costs, development costs, medicine costs, staff salaries, and effectiveness estimates.

### Data Collection and Sample Sizes

In this cRCT (described in full elsewhere [7]), eight rural district hospitals in four provinces in Kenya were randomized into four full and four partial intervention hospitals, hereafter termed intervention and control hospitals [7]. Resource use data were collected via clinical record reviews conducted at baseline and at 6-monthly intervals over an 18-mo period (four surveys in total). During each survey, these reviews were conducted on 400 randomly selected pediatric admissions. Admissions were included if children, aged between 2 and 59 mo, were admitted for acute illnesses during the preceding 6-mo time period. The total sample included 6,199 and 5,115 record reviews of pediatric admissions for intervention and control hospitals, respectively. The clinical performance indicators used as the measure of effectiveness in this analysis were extracted from 1,158 and 1,157 records at 18 mo post implementation in the intervention and control hospitals, respectively.

### The Intervention

The intervention was a package of care intervention that was delivered in the form of evidence-based CPG dissemination [8], health worker training, job aids, follow-up supervision, and local (health facility) facilitation by a nurse or diploma level clinician [7],[9]. The role of the local facilitator was to offer local oversight and on-site problem solving to support facilities implementing the intervention. The training course was developed from the existing World Health Organization (WHO) Emergency Triage, Assessment and Treatment (ETAT) course with the addition of new materials on newborn resuscitation and case management of common causes of serious illness in the newborn or child and with the CPGs spanned: emergency pediatric care, malaria, pneumonia, asthma, diarrhea and dehydration, meningitis, malnutrition, and neonatal care [8]. This new training was therefore given the name “Emergency Triage Assessment and Treatment Plus Admission Care (ETAT+).”

In intervention hospitals, the intervention was delivered over 18 mo as a combination of ETAT+ training for health care workers conducted over 5.5 d, dissemination of CPG booklets, job aids, and pediatric admission record (PAR) forms [7]. The PAR is a structured form used by clinicians to document key symptoms and signs of a sick child's clinical information on admission [14]. This was followed by 2–3-monthly supervisory visits that sometimes included short, ad hoc follow-up training and appointment of a local facilitator in each facility linked to hospital supervisors by regular phone calls from the study team [9],[15]. Results and feedback reports of the surveys conducted in the facilities were disseminated in face-to-face meetings in intervention hospitals.

In the control hospitals, a partial version of the intervention was delivered in the form of CPG booklet distribution, a 1.5-d seminar, and provision of written survey feedback based on written reports only. Control hospitals did not receive any follow-up supervisory support or local facilitation.

### Evaluating Effectiveness

We used process indicators of quality of care to estimate the effectiveness of the intervention. In total 14 pre-specified indicators that span three broad areas were considered as primary outcomes [7]: assessment of a severely ill child, therapeutic care, and supportive care on admission. These indicators cover the diseases (malaria, pneumonia, and diarrhea/dehydration) that result in 60% of inpatient deaths in children under five in Kenya. Effectiveness was then estimated as the between-group (intervention and control) difference using logistic or linear regression analyses for each of the 14 process measures while adjusting for hospital-level covariates (all-cause pediatric mortality, malaria transmission, and hospital size). This procedure is described in detail elsewhere [7]. Further details of the effectiveness analysis are also provided in Figure S1.

### Evaluating Costs

Costs were categorized as intervention development, intervention implementation, and inpatient pediatric treatment costs. The latter were included in order to capture any change in resource use associated with the implementation of best practice pediatric care. Costs were summed across all categories to obtain the total cost per hospital and per hospital admission in intervention and control hospitals. Each cost category is further described in the following sections. Costs were collected using clinical and accounting record reviews and interviews with those involved in developing and implementing the intervention.

### Guideline Development Costs

Development costs included the staff costs incurred in the development of ETAT+ guidelines and training, the costs of course training materials, and costs of organizing and running meetings and workshops. Staff costs were calculated by interviewing key staff involved in guideline development in order to estimate the amount of time spent on these activities. The opportunity cost of this time was then assumed to be equivalent to the associated cost of employment. The costs of course training materials were assumed to be equivalent to the market prices of these items. Development costs were annualized over 4 y, which was assumed to be the useful life of the clinical guidelines.

### Guideline Implementation Costs

Implementation costs included the costs of initial ETAT+ training of health workers, follow-up training, supervisory visits and phone calls, feedback meetings, and on-site local facilitator costs. The opportunity costs of resources used in these activities, e.g., staff time used in attending trainings, were evaluated by estimating the number of days spent at each training workshop and calculating the costs on the basis of the equivalent cost of employment. The costs of the initial training were considered to be capital costs as the effects of the training were expected to be realized over a period of more than 1 y. These costs were annualized over a useful life of 2.5 y, which was the length of time over which the practice change effects were seen to be sustained [7]. Follow-up activities and supervision were considered to be recurrent costs.

### Treatment Costs

Treatment costs were computed as the sum of “hotel,” medicine, and laboratory costs per admission. Resource use data for patient length of stay in hospital, medicines, and laboratory tests were collected from patient clinical records. Estimates of the utilization of these resources were then multiplied against the unit cost of each item. Per diem “hotel” unit costs were derived from the WHO, “Choosing Interventions that are Cost Effective” (WHO-CHOICE) estimates and recent work on the economic costs of inpatient care in Kenya [16],[17]. Medicine unit costs were derived from 2009 market prices while unit costs of diagnostic tests were based on non-profit cost recovery prices from a Kenyan district hospital [16]. Given the skewed nature of cost data, treatment costs are presented as both means (with confidence intervals) and medians (with interquartile ranges).

### Evaluating Cost Effectiveness

The cost-effectiveness analysis compared the implementation of the ETAT+ strategy as delivered in the intervention hospitals with the partial intervention as delivered in the control hospitals. The partial intervention was chosen as a comparator because it mirrors practice that would be considered a basic, standard approach to dissemination of guidelines that does not typically include active follow-up or supervision and for ethical reasons (withholding new national guidelines was deemed unreasonable). While “no intervention” is an alternative counterfactual, this assumes, somewhat unrealistically to us, that no national or international body will produce guidelines or disseminate them or make attempts to improve poor hospital services.

The summary measure of effect was the mean of the adjusted differences between control and intervention hospitals at 18 mo. This was calculated as the mean percentage improvement in the 14 process of care indicators in intervention compared to control hospitals (Equation 1), with 95% CIs obtained by bootstrapping with 2000 iterations.
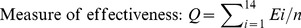
(1)Where: *Q*, mean percentage improvement in process of care; *Ei*, adjusted difference of each process of care between control and intervention hospitals at 18 mo; *n*, number of processes of care.

### Assessing Cost Effectiveness

The incremental cost-effectiveness ratio (ICER) was defined as the incremental cost per percentage gain in mean quality based on the 14 indicators. This is the ratio of the difference in the total admission cost per child between intervention and control hospitals, and the difference in mean quality improvement (Equation 2).
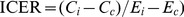
(2)Where: *C*_i_, *c*hild admission costs in intervention hospitals; *C_c_*, *c*hild admission costs in control hospitals; *E*_i_, percentage improvement in process measures of quality in intervention hospital; *E_c_*, percentage improvement in process measures of quality in control hospitals.

The cRCT was not designed to examine effects on health outcomes, therefore we explored the potential incremental cost per DALY averted on the basis of conservative assumptions of the effect of improving quality of care on inpatient childhood mortality. We assumed relative reductions in the mortality rate of between 1% and 10%, equivalent to absolute reductions of between 0.07% and 0.7% with median inpatient mortality, derived from the eight hospitals, equal to 7%. The proportion of lives saved from the respective diseases (malaria, pneumonia, and diarrhea) were assumed to be equivalent to the proportions of the contribution of each of these diseases to under-five childhood deaths in Kenya [18]. In this “what-if” analysis, the intervention was compared to common practice where guidelines are developed and disseminated with no accompanying training and/or follow-up supervision. DALYs were calculated using standard methods [19]. DALYs are generic measures of health outcomes derived by adding the years of life lost due to disease (YLL) and the years of life lived with disability (YLD) [19]–[21]. DALYs were calculated using a discount rate of 3%, age weighting and disability weights for malaria episode (0.19), lower respiratory infection episode (0.28), and diarrheal diseases (0.11) [22]. Separate DALY calculations for each of these diseases where made and summed up to yield total DALYs averted by the intervention.

### Total Costs of Scale-up

Kenya has 121 hospital facilities with estimated median annual pediatric admissions of 2,000 per facility across this group, representing a total for pediatric admissions of 242,000 per annum (there are a larger number of smaller hospitals not considered in this analysis). We estimated the cost of scaling up this intervention with a number of assumptions: (1) Development costs do not vary with scale-up; given that they are only incurred once, they are not a function of the scale of the intervention; (2) That training, supervision, and follow-up costs (implementation) vary as a function of the number of hospitals; (3) That treatment costs vary as a function of the number of pediatric admissions; (4)That the intervention would reach all 121 hospitals when at scale. It is however difficult to estimate potential economies of scale and scope, for example for supervision, that might lessen costs or specific, higher, travel costs for hard to reach areas during scale up. Given the skewness of treatment costs, their scale-up component costs were calculated on the log-scale and then back transformed to the original scale.

### Sensitivity Analysis

Uncertainty was addressed by specifying distributions around cost and effectiveness parameters and conducting probabilistic sensitivity analysis using Crystal Ball software (Decisioneering). Triangular distributions with plausible ranges were fitted around the effectiveness estimate, development costs, salaries, medicines, and “hotel” components of costs (Table 1). Intervention effectiveness was varied to reflect the range of process of care improvements between intervention and control hospitals across the 14 indicators; from 3.54%, to 52.10%, the smallest and greatest reported difference between control and intervention hospitals' indicators. Development costs were varied to reflect a scenario where a “ready made” intervention was adopted hence having zero intervention costs, and a scenario where the full development costs were incurred. The “hotel” unit cost estimate used in the base case, which was also used to compute the lower range in the sensitivity analysis, was the WHO-CHOICE estimate for district hospitals in Kenya, inflated to 2009 (US$6.96 per day) [23], while the upper range was computed from an estimate from a Kenyan study inflated to 2009 (US$15.05 per day) [24]. The lower range of salary costs was derived by assuming the intervention implementers were compensated at government of Kenya salary scales while the upper limit, which was also the estimate used in base case, assumed that intervention implementers were compensated at the salary scales of the research organization that implemented the intervention. The range of medicine costs was derived from variations in market prices in the 2010 Kenya drug prices catalogue. Confidence intervals around the mean ICER were derived from 1,000 Monte Carlo simulations.

**Table 1:** Parameter ranges and distributions.

Parameter (Costs per Child Admission)	Base Case (US$)	Range (US$)	Distribution
Full intervention development costs	8.11	0–8.11	Triangular
Partial intervention development costs	4.95	0–4.95	Triangular
Full intervention salary costs	12.46	11.42–12.46	Triangular
Full intervention hotel costs	20.68	20.68–39.93	Triangular
Full intervention medicine costs	2.30	0.66–8.06	Triangular
Partial Intervention salary costs	3.65	1.67–3.65	Triangular
Partial intervention hotel costs	20.15	20.15–38.89	Triangular
Partial intervention medicine costs	1.74	0.50–6.09	Triangular
Intervention Effectiveness	25.01	3.54–52.10	Triangular

## Results

### Changes in Process of Care Measures

The mean of the adjusted differences of the 14 process measures between control and intervention hospitals was 25.01% (95% CI 17.87%–32.18%). The findings of performance changes across all process measures in both control and intervention hospitals are presented in Table 2.

**Table 2:** Average performance in control and intervention hospitals at baseline and 18 mo follow-up and adjusted difference (95% CI) at 18 mo.

Indicator of Quality of Care	Intervention		Control		Adjusted Difference between Groups at 18 mo (%)^a^	95% CI	
	Survey 1	Survey 4	Survey 1	Survey 4			
**Process indicators**							
**Child's weight documented**	59.30	84.50	21.00	63.20	22.80	−4.05	49.70
**Child's temperature documented**	11.90	71.90	25.10	46.60	26.50	−4.49	57.50
**Average assessment score**	24.00	94.00	32.00	65.00	29.00	5.00	54.00
**Proportion of pneumonia episodes with a severity classification**	9.29	95.10	14.70	57.00	38.57	9.87	67.30
**Proportion of gentamicin prescriptions with once daily dose**	1.85	89.20	3.54	74.40	17.05	8.04	26.10
**Proportion of gentamicin prescriptions with daily dose <4 mg/kg**	24.90	2.16	23.40	8.99	6.77	−11.90	−1.59
**Proportion of gentamicin prescriptions with daily dose** **≥10 mg** **/kg**	3.78	6.25	7.15	9.82	3.54	−11.10	4.00
**Proportion with adequate oxygen prescriptions**	0.00	37.00	0.00	2.31	35.10	7.32	62.80
**Proportion of malaria episodes with a severity classification**	10.10	92.50	2.48	41.10	52.10	26.20	78.00
**Proportion of severe malaria with quinine loading**	4.20	91.90	14.80	66.70	26.30	−3.66	56.30
**Proportion of severe malaria with twice daily quinine maintenance dose**	0.39	87.80	9.95	45.70	42.60	25.10	60.20
**Proportion of severe malaria with quinine daily dose** ≥**40 mg/kg**	7.33	1.02	14.10	7.46	6.53	−12.90	−0.20
**Proportion of dehydration episodes with a severity classification**	52.40	98.30	60.50	84.80	14.40	4.27	24.60
**Correct intravenous fluid prescription**	7.32	67.20	15.00	40.60	29.90	10.90	48.90
**Mean change in quality of care**	13.79	58.98	15.15	39.24	**25.01**	17.87	32.18

a Adjusted difference between intervention arms obtained from linear or logistic regression analysis of hospital summary data adjusting for child's sex and hospital factors (size, malaria endemicity, HIV prevalence, all cause mortality).

### Intervention Costs

Total intervention costs and admission costs per child in intervention and control hospitals are presented in Table 3. An average of 32 health workers underwent the initial ETAT+ training at a cost of US$8,069.32 per intervention hospital or US$252.16 per trainee. Follow-up training, supervision, and local facilitator costs were 19.89% of total intervention costs in intervention hospitals. The annual costs of a local facilitator per facility were US$5,697.87, 5.62% of total intervention costs in intervention hospitals.

**Table 3:** Summary of intervention costs.

Cost Items	Intervention Hospitals			Control Hospitals		As Percent of Total Intervention Costs
	Cost per Hospital US$	Cost per Patient US$^a^	As Percent of Total Intervention Costs	Cost per Hospital US$	Cost per Patient US$	
**Guideline development costs**						
Development costs	16,227.46	8.11	15.98	9,898.29	4.95	15.93
Training material costs	692.92	0.35	0.69	0.00	0.00	0.00
**Total start-up costs**	16,920.39	8.46	16.67	9,898.29	4.95	15.93
**Implementation costs**						
Initial training	8,069.32	4.03	7.94	2,017.33	1.01	3.25
**Follow-up activities**						
Follow-up trainings	4,348.05	2.17	4.28	0.00	0.00	0.00
Local facilitator costs	5,697.87	2.85	5.62	0.00	0.00	0.00
Supervision costs	10,135.50	5.07	9.99	0.00	0.00	0.00
**Total follow-up costs**	20,181.42	10.09	19.89	0.00	0.00	0.00
**Total implementation costs**	28,250.73	14.13	27.85	2,017.33	1.01	3.25
**Start-up and implementation**	45,171.11	22.59	44.52	11,915.62	5.96	19.19
**Treatment costs**						
**Hotel costs**	45,080	22.54	44.42	41,800	20.90	67.28
**Drug costs**	5,080	2.51	4.95	3,600	1.80	5.80
**Lab costs**	11,260	5.63	11.10	6,660	3.33	10.72
**Total treatment cost**	56,304.79	28.15	55.48	50,202.30	25.10	80.81
**Total costs**	101,475.90	50.74	—	62,117.92	31.06	—

a Obtained by dividing the total cost per hospital by the estimated number of annual admissions for children under five per hospital (2,000).

### Treatment Costs

An ordinary linear (OLS) regression of (log transformed) treatment costs revealed that costs did not significantly vary with child diagnosis, hospital, and time (i.e., across the four surveys) (unpublished data). We therefore pooled treatment costs across surveys and diagnoses within each study arm to increase sample sizes. The mean and median treatment costs were US$28.15 (95% CI 27.61–28.70) and US$22.47 (interquartile range [IQR] 14.33–32.78), respectively, in intervention hospitals and US$25.10 (95% CI 24.56–25.65) and US$19.25 (IQR 13.01–29.04) in control hospitals. “Hotel” costs were the key driver of treatment costs and contributed between 73.18% and 79.98% of treatment costs. Treatment costs disaggregated by category are presented in Table S1, while treatment costs per admission episode are presented in Table S2.

### Incremental Costs, Effects, and Cost-Effectiveness Analysis

The incremental cost per admission in intervention hospitals compared to control hospitals was US$19.68 (95% CI 5.31–31.92). The incremental cost per percentage improvement in quality of care was US$0.79 (95% CI 0.19–2.31) per child admission. These results are presented in Table 4.

**Table 4:** Cost-effectiveness analysis.

Strategy	Mean Admission Costs per Child US$ (95% CI)	Incremental Cost US$ (95% CI)	Incremental Effects (Percent Change in Quality of Care) (95% CI)	ICER (95% CI)
**Partial intervention**	31.06 (30.67–47.18)	—	—	—
**Full Intervention**	50.74 (49.26–67.06)	19.68 (5.31–31.92)	25.01% (17.87–32.18)	0.79 (0.19–2.31)

### Estimated Costs of Scale-up and Budget Impact

For an estimated coverage of 121 district hospitals and 242,000 annual under-five admissions, the estimated costs of scale-up were found to be US$3,559,328.78. This amount is estimated to be equivalent to 0.60% of the estimated 2010 annual budget for formal provision of care to children under five in Kenya (Table 5).

**Table 5:** Total costs of scale-up.

Description	Full Intervention US$	Partial Intervention US$
***n*** ** district hospitals**	121	121
**Estimated annual pediatric admission to district hospitals in Kenya**	242,000	242,000
**Costs of national scale-up**	3559328.78	271386.32
**Budget for provision of under-five child health services in Kenya in 2010** a	572,000,000	572,000,000
**Impact of scaling up ETAT+ on the annual child health budget**	0.60%	0.06%

a Estimates of annual budget (2010) for provision of care to children under five derived from the Kenya national health sector strategic plan 2 (NHSSP II).

### Incremental Cost per DALY Estimates Given Probable Reductions in Mortality

**Figure 1:**
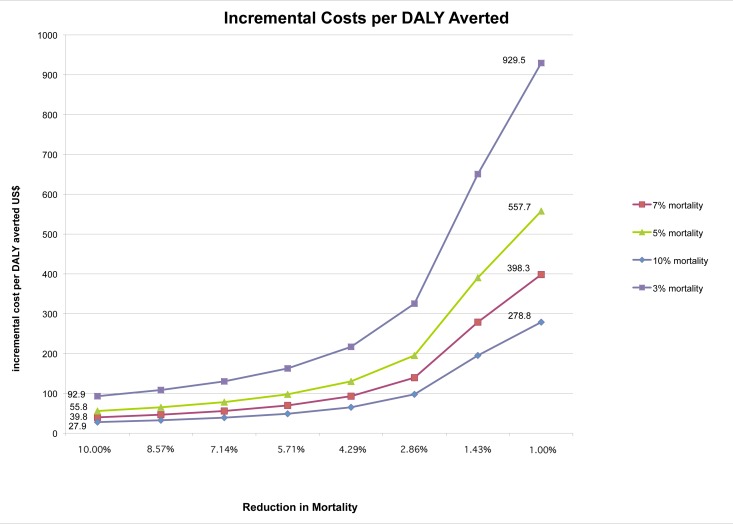
Relationship between reductions in inpatient mortality and the incremental cost per DALY averted.

The mean baseline inpatient child mortality rate in the eight hospitals was 7% [7]. Assuming the intervention produces a 1%–10% relative reduction in this mortality rate (absolute reductions between 0.07% and 0.7%), the incremental cost per DALY averted would range between US$398.3 and US$39.8, respectively. Figure 1 explores the relationship between reduction in mortality and intervention cost effectiveness at different baseline mortality rates while Table 6 compares the range of potential ICERs with those from other key child health interventions that are considered cost effective.

**Table 6:** Cost per DALY averted estimates of comparable child health interventions.

Child Health Intervention	Incremental Cost per DALY Averted US$^a^
Expanded immunization programme [39]	13.0–26.1
Hemophilus Influenzae vaccine (Hib) [46]	32.4–78.6
Provision of insecticide treated nets (ITNs) [38]	34.6–154.8
Improving inpatient care of very sick children (assuming between 10% and 1% reduction in baseline inpatient mortality rate)	39.8–398.3
Integrated Management of Childhood Illnesses (IMCI) [47]	47.1–157.1
Pneumococcal conjugate vaccine [48]	71.1–230.7
Oral rehydration therapy (ORT) [39]	172.2–3352.0
Measles immunization [39]	335.2–5954.1
Breast feeding promotion programmes [39]	687.4–2609.9

a ICER values adjusted to 2009 values using GDP deflators.

### Sensitivity Analysis

**Figure 2:**
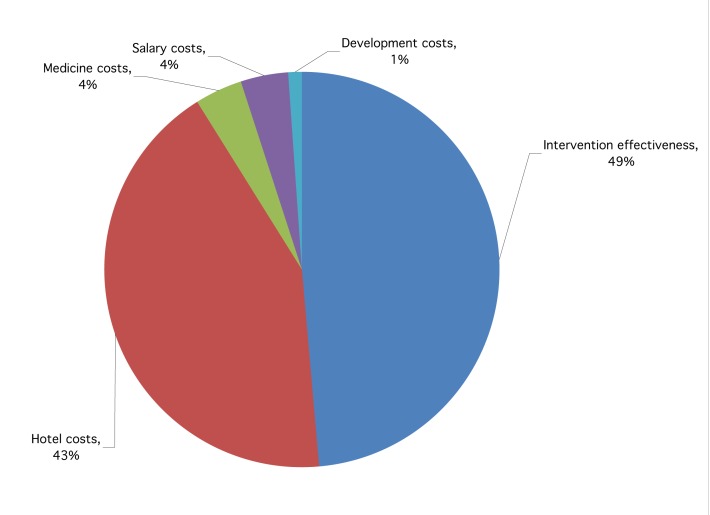
Parameter contribution to ICER variance.

The incremental cost-effectiveness ratios were robust to changes in most of the variables included in the sensitivity analysis. Four factors (intervention effectiveness, hotel costs, medicine costs, and staff salaries) contributed 99% of the total variance in the ICER (Figure 2). The major contributors to this variance were intervention effectiveness (49%) and hotel costs (43%). Salary and medicine costs contributed to 4% of ICER variance each, while development costs contributed 1% of the variation. The higher the intervention effectiveness, the lower the ICER (increased intervention cost effectiveness), while the higher the costs (hotel, salary, medicine, development costs) the higher the ICER (reduced intervention cost effectiveness).

## Discussion

This analysis compares the costs and effects of a guideline-based intervention aimed at improving the quality of care of children admitted to district hospitals in Kenya. In analyzing the costs, we included the costs of developing clinical guidelines that are often left out in such analyses [25]. Our analysis revealed that these costs formed 16% of the overall intervention costs, suggesting that this component is an important cost driver that should not be ignored. The development costs are however minimal at scale-up, given that they are incurred once and do not vary with the scale of the intervention. The average training cost per health worker was US$252.16, which is significantly lower than training costs for similar interventions such as Integrated Management of Childhood Illness (IMCI) where the reported median cost was US$633 [26]. One of the unique components of this intervention was the appointment of a local facilitator in intervention hospitals. The annual cost of this facilitator per facility was US$5,698. The local facilitator worked at the facility for the entire intervention period. This provided continuity and helped to keep the “quality agenda” on the table [27].

There are many challenges in undertaking cost-effectiveness analyses of interventions targeting hospitals and multiple diseases. Basic challenges include the lack of high quality data on hotel costs. These represent between 73% and 80% of total treatment costs for children admitted with common diseases even though inpatient stays are typically 3 d or less. We used the WHO-CHOICE hotel cost estimates applicable to district hospitals in Kenya. In effect, the use of these data amounts to an assumption that there are no major differences in the intensity of staffing per patient. While this assumption was justified as we did not expect that our intervention would require different levels of health worker input within the different hospital settings, we acknowledge that poor primary data is a potential shortcoming and suggest that addressing this information gap is a priority.

Specifying summary measures that capture intervention effects is also a challenge [28]. We used a mean percentage improvement in quality assessed across 14 indicators for the three major causes of admission and inpatient death in children [7]. By this measure, intervention hospitals outperformed control hospitals by 25%. However, such a measure gives equal weight to each indicator, with which some might disagree. This assumption is, however, consistent with findings from an international Delphi study conducted with pediatric experts where the respondents rated these 14 process of care indicators as having similar priority for improvement [29]. It can be argued however that certain process measures, for example those targeting appropriate dosing of medicines, are more important than others, such as processes that target patient assessment. Despite the lack of an agreed weighting for these processes, we explored a weighting procedure (unpublished data) that gives higher weight to treatment processes (weight 3), followed by diagnosis processes (weight 2), and lastly assessment processes (weight 1). The resultant effect size (24.0%) was not significantly different from the unweighted estimate (25.01%) and fell within the range used in the sensitivity analysis. The effectiveness measure also fails to capture potentially important additional improvements resulting from intervention, for example improved organization of care, better local resource mobilization [7], more rational use of antibiotics [30], and positive effects on staff morale [15]. Conversely the measure does not capture potential declines in quality in other areas that are not the focus of intervention. Methodological research is thus needed to optimize effect measures for complex interventions targeting improvements in hospital care for multiple diseases.

While acknowledging these limitations our findings suggest an additional cost of US$0.79 per child admitted to achieve a one percentage point improvement in this summary quality measure. The probabilistic sensitivity analysis reveals that hotel costs and intervention effectiveness contributed to more than 80% of the variation in this ICER. This finding has two implications: (1) The sensitivity analysis underlines the effects of poor data on hotel costs and methodological deficiencies in computing a summary measure of quality improvement; (2) Hotel costs (being key drivers of treatment costs) and intervention effectiveness have a significant impact on the cost effectiveness of this quality of care intervention.

Translating improvements in process measures into improved health status outcomes is problematic and the cluster randomized trial was not designed to measure effects on inpatient child mortality [31]. However, to provide some means to consider the potential value of the intervention in terms of incremental cost per DALY averted and life years gained, we conducted a simple “what-if” analysis. We considered a reasonably conservative range of relative reductions in baseline mortality of between 1% and 10%, absolute reductions of mortality of 0.7% to 0.07% from a baseline of 7%. This range of mortality reductions is considered conservative when compared to findings suggesting improved case management for common childhood diseases in primary care may result in mortality reduction of 13% in Tanzania [32]. Also, evaluations of quality improvement and safety programmes in developed countries have reported mortality reductions of between 5% and 51% [33]–[37]. The implication is that the cost effectiveness of the interventions is likely to be more favorable than we have suggested. The findings suggest that the incremental cost per DALY averted from scaling up the intervention would vary from US$39.8 to US$398.3. These ICERs compare favorably with other key public health interventions to reduce child mortality considered to be cost effective such as provision of insecticide treated bed nets (US$34.60–US$154.8 per DALY averted) [38] or oral rehydration therapy (ORT) (US$172.2–US$3352.0 per DALY averted) [39]. The intervention is likely most cost effective when hospital mortality is high (Figure 1) and baseline quality of care poor. Such analyses raise the question of whether quality improvement should be targeted at high mortality hospitals. While such simple modeling approaches suggest improving the quality of rural hospital care may be highly cost effective, demonstrating such small reductions in mortality in a randomized controlled trial would be extremely difficult. For example, demonstrating an absolute, inpatient mortality reduction of 0.5% from a baseline of 7% in a typical cluster randomized trial, using conventional values for statistical significance and power, would likely require all 121 Kenyan hospitals to be enrolled, randomized, and evaluated over 1 y [40]. The costs of such a trial would be prohibitively high and would amount to scaling up anyway.

Often the feasibility of scaling up is determined by likely costs. For the multifaceted intervention employed these were estimated to be US$3,559,328 if conducted by non-government personnel and 27% less if by government personnel. This amount can be compared with average annual projected expenditures by the Kenyan government on all care for children under five of US$572 million [41] and on other specific health projects such as the distribution of insecticide treated nets (US$8 million) and prevention of mother to child transmission of HIV (US$6 million) [42]. While the costs of scale-up might be small (approximately 0.6% of the child health budget), scaling up the ETAT+ intervention nationally would either require the child health budget to be increased or that allocation to other areas be reduced by an equivalent amount.

Cost-effectiveness and affordability data are, however, not the only factors that should inform such allocative decisions. Other important considerations may include, equity, likely collateral benefits or adverse effects, and, of course, context and the politics of the day. Unfortunately methods to support and make transparent such complex prioritization decisions remain poorly developed. Advantages of scaling up such an integrated package of interventions encompassed in the ETAT+ strategy include potentially important externalities related to more general health system strengthening and introduction of a culture of improvement [7],[27]. For those in other settings reviewing these results, it should be clear that the greatest apparent cost effectiveness is likely to occur in settings providing, reliably, a minimum set of basic resources but where quality of care, in terms of process, is poor and mortality high. Findings from this work are therefore likely to be generalizable to low-income countries with similar public hospital characteristics, child burden of disease, and comparable quality of delivery of pediatric care as found in hospitals at baseline in Kenya.

Our work adds to a very small body of literature on economic evaluation of quality of care interventions [43]. The few that are available from low-income settings, including evaluations of Integrated Management of Childhood Illness (IMCI), adopted a similar approach [32],[44],[45]. A major limitation of these approaches is that they fail to fully elucidate the value of the intervention to the patients, health workers, and organizations, and by extension to decision makers. Further, because effect measures are unique to the interventions it is not possible to compare results directly with other cost-effectiveness or cost-utility studies. To provide a more explicit framework for comparison requires modeling the link between process measures of improved quality and health outcomes, which would be complex and likely based largely on expert opinion. As an alternative we used an approach that explored potentially plausible overall effects on hospital mortality. This can clearly be challenged but is mainly used for illustration. In the absence of data on mortality effects perhaps a method of valuing quality improvement interventions that encompasses more than health outcomes might be more appropriate and could reflect utility by, for example, eliciting society's preferences.

### Conclusion

This analysis has shown that the improvement in quality of care attributed to the ETAT+ strategy (7) is associated with additional costs that are affordable to low-income countries like Kenya. The intervention may be relatively cost effective compared with standard care if the improvements observed are associated with reasonably conservative reductions in inpatient child mortality. The absolute costs for scaling up are comparable to or lower than costs of other, major child health interventions. As increasing focus is being given to strengthening health systems there would therefore appear to be a reasonably strong case for scaling up this intervention that improves service provision in rural hospitals for the major causes of child mortality in Kenya. This work also highlights the need for methodological developments in the economic analysis of complex, system-level interventions. These results are likely to be most usefully generalized to low-income countries beyond Kenya with similar facilities, burden of child mortality, and comparable or worse quality of pediatric care in hospitals.

## Supporting Information

Figure S1 Two-stage analysis plan for intervention effectiveness based on Hayes and Moulton [49].
(TIF)

Table S1 Treatment costs per admission.
(DOC)

Table S2 Admission treatment costs per diagnosis.
(DOC)

## Abbreviations

CPG: clinical practice guideline
cRCT: cluster randomized trial
DALY: disability adjusted life years
ETAT+: Emergency Triage and Treatment Plus
ICER: incremental cost-effectiveness ratio
WHO: World Health Organization
