# Supplementary figures and images for: A Multifaceted Intervention to Improve the Quality of Care of Children in District Hospitals in Kenya: A Cost-Effectiveness Analysis
Source: PLoS Med. 2012 Jun 12;9(6):e1001238. doi: 10.1371/journal.pmed.1001238 (PMC3373608; doi:10.1371/journal.pmed.1001238)

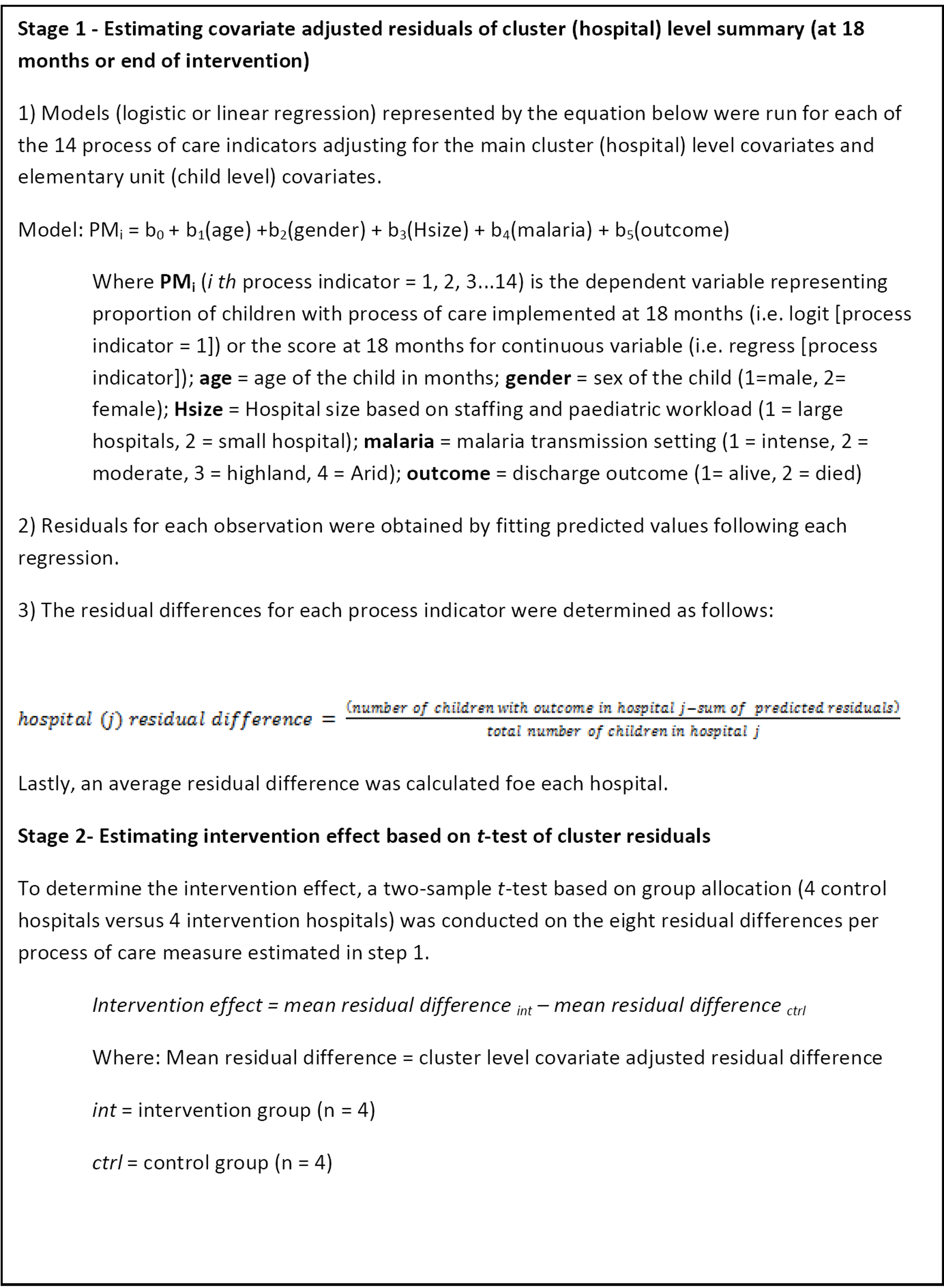

Supplement: Figure S1 — Two-stage analysis plan for intervention effectiveness based on Hayes and Moulton [49]. (TIF) [file pmed.1001238.s001.tif]
