# Supplementary material for: A Multifaceted Intervention to Improve the Quality of Care of Children in District Hospitals in Kenya: A Cost-Effectiveness Analysis
Source: PLoS Med. 2012 Jun 12;9(6):e1001238. doi: 10.1371/journal.pmed.1001238 (PMC3373608; doi:10.1371/journal.pmed.1001238)
Supplement: Table S1 — Treatment costs per admission. (DOC) [file pmed.1001238.s002.doc]

**Table S1: Treatment Costs per Admission**

|  | **intervention hospitals** | | | | **control hospitals** | | | |
| --- | --- | --- | --- | --- | --- | --- | --- | --- |
| **Cost Items** | **N** | **median cost US$(IQR)** | **Mean Cost US$ (95%CI)** | **As a % of**  **Treatment Costs** | **n** | **median cost US$(IQR)** | **Mean Cost US$ (95%CI)** | **As a % of**  **Treatment Costs** |
| **Hotel Costs** | 4,963 | 18.64 (9.55-23.88) | 22.54 (21.99-23.09) | 73.18 % | 4,460 | 14.33 (9.55-23.88) | 20.90 (20.36-21.44) | 79.98 % |
| **Drug Costs** | 5,514 | 1.68 (0.72-3.00) | 2.51 (2.43-2.59) | 8.15 % | 4,460 | 1.11 (0.43-2.29) | 1.80 (1.73-1.86) | 6.89 % |
| **Lab Costs** | 6,199 | 3.36 (0.00-3.36) | 5.63 (5.52-5.75) | 18.28 % | 5,115 | 3.36 (0.00-3.36) | 3.33 (3.23-3.44) | 12.74 % |
| **Treatment Costs** | 5,678 | 22.47 (14.33-32.78) | 28.15 (27.61-28.70) |  | 4,691 | 19.25 (13.01-29.04) | 25.10 (24.56-25.65) |  |
