# Supplementary material for: A Multifaceted Intervention to Improve the Quality of Care of Children in District Hospitals in Kenya: A Cost-Effectiveness Analysis
Source: PLoS Med. 2012 Jun 12;9(6):e1001238. doi: 10.1371/journal.pmed.1001238 (PMC3373608; doi:10.1371/journal.pmed.1001238)
Supplement: Table S2 — Admission treatment costs per diagnosis. (DOC) [file pmed.1001238.s003.doc]

**Table S2: Admission Treatment Costs per Diagnosis**

|  | **Intervention Hospitals** | | **Control Hospitals** | |
| --- | --- | --- | --- | --- |
| **Diagnosis** | **n** | **Mean Cost per admission US $(95%CI)** | **n** | **Mean Cost per admission US $(95%CI)** |
| **Malaria** | 1,017 | 26.06 (24.70-27.41) | 1,042 | 21.28 (20.34-22.23) |
| **Pneumonia** | 541 | 26.04 (24.57-27.51) | 622 | 26.68 (25.07-28.29) |
| **Diarrhea & Dehydration** | 225 | 24.62 (22.50-26.74) | 223 | 25.63 (23.21-28.05) |
| **Pneumonia & Malaria** | 1,262 | 26.31 (25.41-27.21) | 738 | 24.78 (23.39-26.16) |
| **Pneumonia & Diarrhea** | 76 | 27.98 (23.33-32.63) | 80 | 26.65 (22.11-31.19) |
| **Malaria & Diarrhea** | 428 | 24.13 (22.53-25.73) | 364 | 22.10 (20.64-23.57) |
| **Malaria & Pneumonia & Diarrhea/Dehydration** | 222 | 26.58 (24.34-28.82) | 82 | 25.60 (21.37-29.83) |
